# Study on the coupling relationship between urban resilience and urbanization quality—A case study of 14 cities of Liaoning Province in China

**DOI:** 10.1371/journal.pone.0244024

**Published:** 2021-01-04

**Authors:** Yanpeng Gao, Wenjun Chen

**Affiliations:** Jiangho School of Architecture, Northeastern University, Shenyang, Liaoning Province, China; Institute of Geographic Sciences and Natural Resources Research (IGSNRR), Chinese Academy of Sciences (CAS), CHINA

## Abstract

In this paper, entropy, coupling coordination degree, spatial auto-analysis, LISA time path, and other methods have been used to analyze the coupling coordination degree of urban resilience and urbanization quality of 14 cities in Liaoning Province from 2009 to 2019. The results show that: 1. The number of highly resilient cities accounts for 14.3% of the total number of cities in Liaoning Province, and the overall resilience degree is low; the spatial distribution shrinks along the Shenyang–Dalian Economic Belt toward both sides, with obvious “core-margin” characteristics. 2. The average score of urbanization quality increased from 0.0574 to 0.0966, showing a fluctuating upward trend; the regional difference was significant, and the “dual-core” characteristic was prominent. 3. During the study period, the 14 cities of Liaoning Province were in a state of imbalance, and there was a positive correlation between the coupling degree and the coordination degree. Moran’s I decreased from 0.237 to 0.220 and the spatial agglomeration characteristics also weakened. Further analysis of the spatial and temporal linkage characteristics of the coupling relationship shows that the relative length of LISA time path presents characteristics of protrusion in the central region and shrinkage on the East and West sides, and the curvature presents characteristics that are smaller in the North and larger in the South.

## 1. Introduction

As a basic attribute of the ability to handle urban risks, urban resilience is an emerging field in urban governance research. The definition of “resilience” originated from the engineering concept of “returning to the original state”. It was first proposed in 1973 by Holling, a Canadian ecologist with application to system ecology. Later, this concept was applied to many other unrelated fields such as economics, sociology, and management [1]. Narrowly speaking, urban resilience is the ability of an urban system to handle sudden and destructive natural phenomena, and to adopt disaster prevention measures for control and resistance; broadly speaking, urban resilience also includes economic resilience, social resilience, engineering resilience, ecological resilience, and other multidimensional perspectives.

The dynamic coupling relationship between urban resilience and urbanization is reflected in the fact that both consist of economic, social, ecological, and other urban subsystems. These comprehensive systems are the main driving force of industrial economy; they represent the transformation of urban functions into important manifestations, and their culmination is sustainable development. They are also an important part of China's new-type urbanization, and have had an impact on urban climate, land use, and element flow [2–5].

The rapid development of urbanization not only brings new development opportunities for a city and promotes resilience. However, the rapid development of urbanization has a high degree of dependence on resources, resulting in low resource utilization, and the mismatch between urban infrastructure construction and development level, which makes it difficult to meet the needs of residents and hinders the development of urban disaster resistance. A high degree of urban resilience provides a safe and stable operating environment for the city and promotes the process of urbanization; a low degree of urban resilience leads to a high probability of the occurrence of urban risks, and a weak capacity in urban absorption and recovery, which in turn severely restricts the process of urbanization.

In recent years, many scholars in China and abroad have studied urban resilience from both the macro and micro perspectives. From a macro perspective focuses on concepts and characteristics, such as the evolution of the concept of resilience [6], research framework [7, 8], action mechanism [9] and characteristic attributes [10], etc., to fully grasp urban resilience Definition and development direction; the micro perspective is mostly based on the risks that cities may cope with, such as comprehensive quantitative evaluation of urban disaster resilience to earthquake disasters [11], extreme weather [12, 13], and flood disasters [14]; or from the basic components build an evaluation system to evaluate urban infrastructure [15, 16] economy [17, 18], society [19], ecology [20–22] and other urban subsystems. Carry out a comprehensive evaluation of the degree of urban development. On the one hand, the research of urbanization lies in how to optimize the development of urbanization. Scholars at home and abroad have conducted research on the types of urbanization models [23], development paths [24, 25], and influence mechanisms [26, 27] and other urbanization construction content, and proposed Policy recommendations to promote the further improvement of the level of urbanization construction; on the other hand, it focuses on the diversified development of urbanization, that is, the cross-extension of urbanization and multidisciplinary. This part of the research mostly focuses on urbanization development and land use [28–30], industrialization [31], ecological environment [32, 33] and other parts, in-depth understanding of the relationship between the two, dynamic characteristics, and spatial characteristics. The research on coupling relationship is mostly used to measure the related influence of two or more systems, so it is often introduced into many fields such as ecology [34, 35], economy [36, 37], geography [38], etc. From the perspective of urban space, the research content focuses on land use [39, 40], urban resources [41], and human settlements [42, 43]. There are few studies on urban resilience and urbanization quality in research on urban resources, transportation infrastructure, and human settlements. To summarize, research on urban resilience and urbanization has relatively matured in their respective fields, however, there have been limited discussions on the coordination relationship between the two, as well as the quantitative research and spatial analysis from the perspective of coupling and coordination.

With the implementation of the Northeast Revitalization Strategy, the urbanization in Liaoning Province has seen rapid and vigorous promotion, leading to the improved economic strength of the province. However, problems such as the inappropriate utilization of land resources and concentration of economic activities in few areas have also hindered the improvement in the quality of urbanization and affected coordinated regional development. Therefore, clarification of the spatial differences and the coupling relationship between urban resilience and urbanization quality are of significant importance in solving the urban development dilemma and the optimization of the economic and social spatial pattern. Based on this, this paper uses entropy, coupling coordination degree model, spatial auto-analysis, and LISA time path methods to carry out the coupling analysis of the urban resilience and urbanization quality of 14 cities in Liaoning Province from 2009 to 2019, so as to provide a theoretical reference data framework for the promotion of good regional development.

## 2. Data sources and research methods

### 2.1 Construction of evaluation indicator system

Urban resilience refers to the ability of urban systems to resist and absorb external adverse impacts and self-repair in the face of natural or man-made disturbance. Previous research done by scholars and the availability of suitable data over the period 2009 to 2019, was utilized to construct an evaluation indicator system from the following four dimensions: economy, society, ecology, and engineering (Table 1). Four indicators of economic resilience were selected from aspects of industrial structure and income; six indicators of societal resilience were selected from aspects of consumption, medical care, education, and insurance; five indicators of ecological resilience were selected from aspects of urban green space area and pollutant emission; and five indicators of engineering resilience were selected from the aspects of road network, pipelines, and energy consumption. This method determines the weight by calculating the information entropy of the indicator. The indicator with a relatively large (small) change has a large (small) weight, which can overcome the arbitrariness of subjective weight.

**Table 1:** Urban resilience evaluation index system and weight.

First-level indicator	First-level indicator weight	Secondary indicators	Secondary index weight
		GDP per city area (yuan/km^2^)	0.0637
Economic resilience	0.3095	GDP per capita in municipal districts (yuan/person)	0.0571
		Proportion of tertiary industries in municipal districts (%)	0.068
		Per capita disposable income of urban residents in municipal districts (yuan/person)	0.0759
		Per capita total social consumer goods in municipal districts (yuan/person)	0.0448
Social resilience	0.2900	Number of beds in medical institutions per 10,000 people in the municipal area (pieces/10,000 people)	0.059
		Number of doctors per 10,000 people in the municipal area (person/10,000 people)	0.0454
		Number of college students per 10,000 people in the municipal area (person/10,000 people)	0.0411
		Urban registered unemployment rate (%)	0.045
		Insurance premium income per capita in the city (yuan/person)	0.0515
		Per capita water supply in municipal districts (m^3^/person)	0.048
		Per capita heat supply in municipal districts (GJ/person)	0.0483
Engineering resilience	0.2712	Per capita road area in municipal districts (m^2^/person)	0.0432
		Density of road network in municipal district (%)	0.0493
		Drainage pipeline density in municipal district (%)	0.0421
		Green coverage rate in built-up area (%)	0.0553
		Per capita green area in municipal districts (m^2^/person)	0.033
Ecological resilience	0.1293	Industrial wastewater discharge per unit GDP (tons/10,000 yuan)	0.0465
		Industrial waste gas emissions per unit GDP (standard cubic meters/yuan)	0.0396
		Industrial solid waste discharge per unit GDP (tons/10,000 yuan)	0.0432

Note: The data in the table is calculated according to the entropy method.

Urbanization refers to the gradual transformation of the population of a country or region from rural to urban along with connected economic and social development parameters, with specific focus on the following characteristics: increasing the proportion of urban population in the total population, industrial restructuring, construction of modern urban infrastructure, and transformation of land function. Based on this, in order to measure the quality of urbanization more comprehensively, the indicator evaluation system was constructed from eight indicators based on population urbanization, economic urbanization, social urbanization, and land urbanization (Table 2).

**Table 2:** Urbanization quality evaluation index system and weight.

First-level indicator	First-level indicator weight	Secondary indicators	Secondary index weight
Population urbanization	0.1809	Urbanization rate (%)	0.0589
		Urban population density (person/km^2^)	0.122
Economic urbanization	0.2271	The proportion of the output value of the secondary and tertiary industries (%)	0.093
		Average salary of employed employees in urban units (yuan/person)	0.1341
Social urbanization	0.2922	Educational financial expenditure (ten thousand yuan)	0.1219
		Financial expenditure on health, social security and social welfare (ten thousand yuan)	0.1703
Land urbanization	0.2998	Proportion of construction land area in urban area (%)	0.1476
		Per capita construction land (m^2^/person)	0.1522

Note: The weight data in the table is calculated according to the entropy method.

### 2.2 Study area

**Fig 1:**
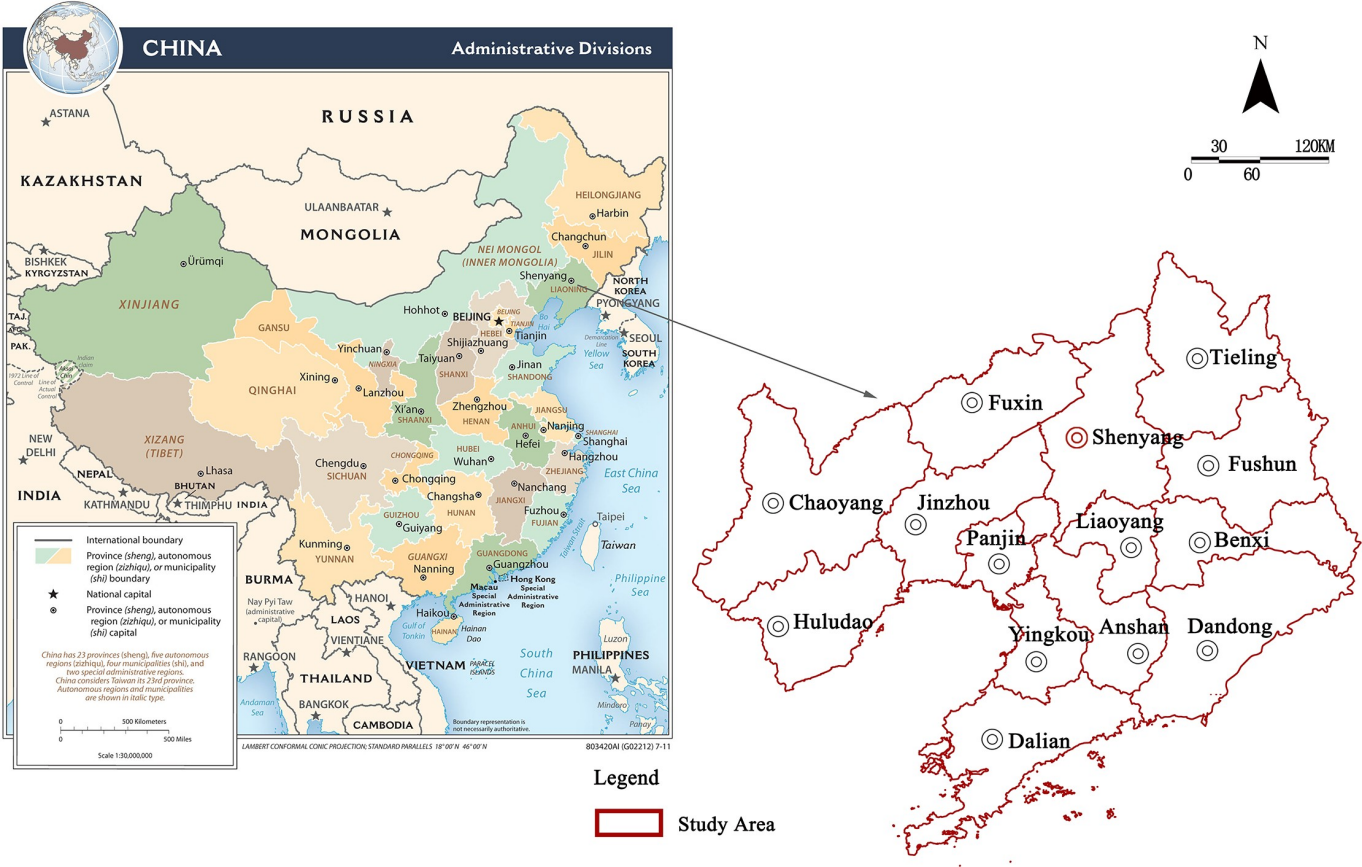
Location map of Liaoning Province (image source: https://www.cia.gov/library/publications/resources/cia-maps-publications/China.html).

The study area of this article is Liaoning Province, a provincial administrative region in China. Liaoning Province is in the southern part of Northeast China, bordering Hebei Province in the southwest, Inner Mongolia Autonomous Region in the northwest, and Jilin Province in the northeast (Fig 1).

### 2.3 Data sources and processing

The data used in this study mainly include geospatial data and social statistical data (Table 3).

**Table 3:** Summary of data and sources used in this article.

	Usage data	Data source
Geospatial data	Satellite remote sensing data of cities and above in Liaoning Province	U.S. NAS Landsat Landsat4-5 in 2008, Langdsat8 satellite remote sensing data in 2020 and ASTER GDEM with 30m resolution
	Administrative boundaries of Liaoning Province	National Earth System Science Data Sharing Platform
Social statistical data	Economic, social, population, and ecological data of cities and above in Liaoning Province from 2010 to 2020 (as of September)	2010–2020 (as of September) Liaoning Province Statistical Yearbook, China Economic Net statistical database and EPS global statistical data/analysis platform, Liaoning Province and above cities at the national economic and social development statistical bulletin 2010–2020 (as of September)

This paper used ENVI 4.7 remote sensing image processing platform to preprocess the image data acquired during the study period, such as radiometric calibration, atmospheric correction, etc., to delineate the relevant data from remote sensing images of Liaoning Province according to administrative boundaries, followed by human-computer interactive interpretation, to extract land information.

Due to the different dimensions of various indicators from different sources, dimensionless standardized formula was used to process the original data. At the same time, some missing data were estimated using the difference value method. It is worth mentioning that the social statistics from 2010–2020 (finally to the second quarter) are selected here to better represent the urban resilience and urbanization quality changes in Liaoning Province from 2009 to 2019.

### 2.4 Research methods

#### 2.4.1 Entropy methods

The Entropy Method is an objective weighting method. Through the correlation between the original data of each indicator, the weighted value for different indicators can be determined, which can objectively reflect the weightage of each indicator and effectively avoid the deviation caused by subjective factors [44].

$$e_{j}=-k\sum_{i=1}^{m}y_{ij}lny_{ij},\;k=1/lnm$$ (1)

$$d_{j}=1-e_{j}$$ (2)

$$W_{j}=d_{j}/\sum_{i=1}^{m}d_{j}$$ (3)

$$U=\sum_{i=1}^{m}y_{ij}W_{j}$$ (4)

Where: *e*_*j*_ is the information entropy; *m* is the number of research units; *k* is the reciprocal of ln *m*; *y*_*ij*_ is the comprehensive score of the *j* th indicator of the *i*th system; *d*_*j*_ is the information utility value; *W*_*j*_ is the weightage of the indicator; and *U* is the comprehensive score.

#### 2.4.2 Coupling coordination degree model

The Coupling Coordination Degree Model is used for analyzing the degree of coordinated development between things. The concept of degree of coupling originates from physics and refers to the interactive influence between two or more systems, which can reflect the degree of interdependence and mutual restrictions between systems [45, 46]. The calculation formula is as follows:

$$C=2\times {\left\{\frac{P_{i}\times R_{i}}{{{(P}_{i}+R_{i})}^{2}}\right\}}^{\frac{1}{2}}$$ (5)

Where: *C* is the coupling degree between urban resilience and urbanization quality, and the value range is [0, 1]; *P*_*i*_ and *R*_*i*_ are the comprehensive evaluation values of urban resilience and urbanization quality, respectively.

While the coupling degree can reflect the degree of interaction between the two, it cannot indicate whether the development of the two is consistent. Therefore, the coordination degree model is introduced to construct the coupling coordination model and have it graded. According to the merger coordination degree, the uniform distribution function method is adopted to classify the coupling coordination degree (Table 4). The calculation formula is as follows:

$$D=\sqrt{C\times T},\;T=\alpha P+\beta R$$ (6)

Where: *D* is the degree of coupling coordination between urban resilience and urbanization quality; *P* and *R* are the evaluation values of urban resilience and urbanization quality, respectively; *α* and *β* are undetermined coefficients, representing the importance of urban resilience and urbanization quality, respectively, where *α* + *β* = 1. As urban resilience and urbanization quality are equally important, the values of *α* and *β* are both 0.5.

**Table 4:** Classification standard of coupling coordination degree.

Coupling coordination degree D value interval	Coordination level	Coupling degree
(0.0~0.1)	1	Extreme imbalance
[0.1~0.2)	2	Serious disorder
[0.2~0.3)	3	Moderate Disorder
[0.3~0.4)	4	Mild disorder
[0.4~0.5)	5	Edge imbalance
[0.5~0.6)	6	Barely coordinated
[0.6~0.7)	7	Primary coordination
[0.7~0.8)	8	Intermediate coordination
[0.8~0.9)	9	Well-coordinated
[0.9~1.0)	10	Quality coordination

#### 2.4.3 Spatial autocorrelation

Spatial Autocorrelation is a method of spatial data analysis that studies whether the data has a correlation relationship in terms of spatial unit. It can be used to explore the degree of spatial agglomeration of the subject reflected by the data [47], which is usually divided into global and local spatial autocorrelation.

$$I=\frac{\sum_{i=1}^{n}\sum_{j=1}^{n}W_{ij}(x_{i}-\bar{x})(x_{j}-\bar{x})}{S^{2}\sum_{i=1}^{n}\sum_{j=1}^{n}W_{ij}}$$ (7)

$$S^{2}=\sum_{i=0}^{n}\left(x_{j}-\bar{x}\right)$$ (8)

Where: *x*_*i*_ and *x*_*j*_ are the variable values of adjacent areas; *W*_*ij*_ is the value of an element of w—the spatial weighted matrix; n is the number of spatial units; the value range of *I* is [–1, 1]. If *I*∈[-1,0], it indicates that there is a negative spatial correlation; if *I*∈[0,1], it indicates that there is a positive spatial correlation.

Local spatial autocorrelation is mainly used to measure the local spatial correlation characteristics between geographical units and their adjacent areas. It is usually represented by LISA indicators [48], and the calculation formula for these is as follows:

$$I_{i}=z_{i}\sum_{j=1,j\neq i}^{n}W_{ij}Z_{ij}$$ (9)

Where: *z*_*i*_ and *z*_*j*_ are geographical units; *i* and *j* attribute values are normalized values of variance; ∑*w*_*ij*_*z*_*ij*_ is the spatial lag vector, and other variables are the same as above.

#### 2.4.4 LISA time path

In terms of LISA time path, we studied the movement of LISA coordinates on a Moran scatter diagram and combined the temporal and spatial properties, thus making the traditionally static LISA dynamic [49–51]. Based on its geometric characteristics, the time path is decomposed into path length and curvature, of which, the relative length can reflect the dynamic characteristics of local spatial structure, and the curvature can show the fluctuation of local spatial structure. The moving path of LISA coordinates in different years can be represented as [(*y*_*i*,*1*_, *yL*_*i*,*1*_),(*y*_*i*,*2*_, *yL*_*i*_,_*2*_), …, (*y*_*i*,*t*_, *yL*_*i*,*t*_)], where *y*_*i*,*t*_ is the standardized value of coupling coordination degree of city *i* in the year *t*, and *yL*_*i*,*t*_ is the spatial lag of city *i* in the year *t*.

$$d=\frac{N\sum_{t=1}^{T-1}d(L_{i,t},L_{i,t+1})}{\sum_{i=1}^{N}\sum_{t=1}^{T-1}d(L_{i,t},L_{i,t+1})}$$ (10)

$$f=\frac{\sum_{t=1}^{T-1}d(L_{i,t},L_{i,t+1})}{d(L_{i,t},L_{i,t+1})}$$ (11)

Where: *d* is the length of time path, and greater the value, stronger the dynamic of local spatial structure; *f* is the curvature of the path, and greater the value, curvier the LISA time path and bigger the fluctuation of local spatial structure; *N* is the number of cities (number); *T* is the time interval (number of years); *d(L*_*i*,*t*_, *L*_*i*,*t+1*_*)* is the moving distance of city *i* from the year *t* to the year of *t+1*.

## 3. Result analysis

### 3.1 Differential characteristics of urban resilience

**Fig 2:**
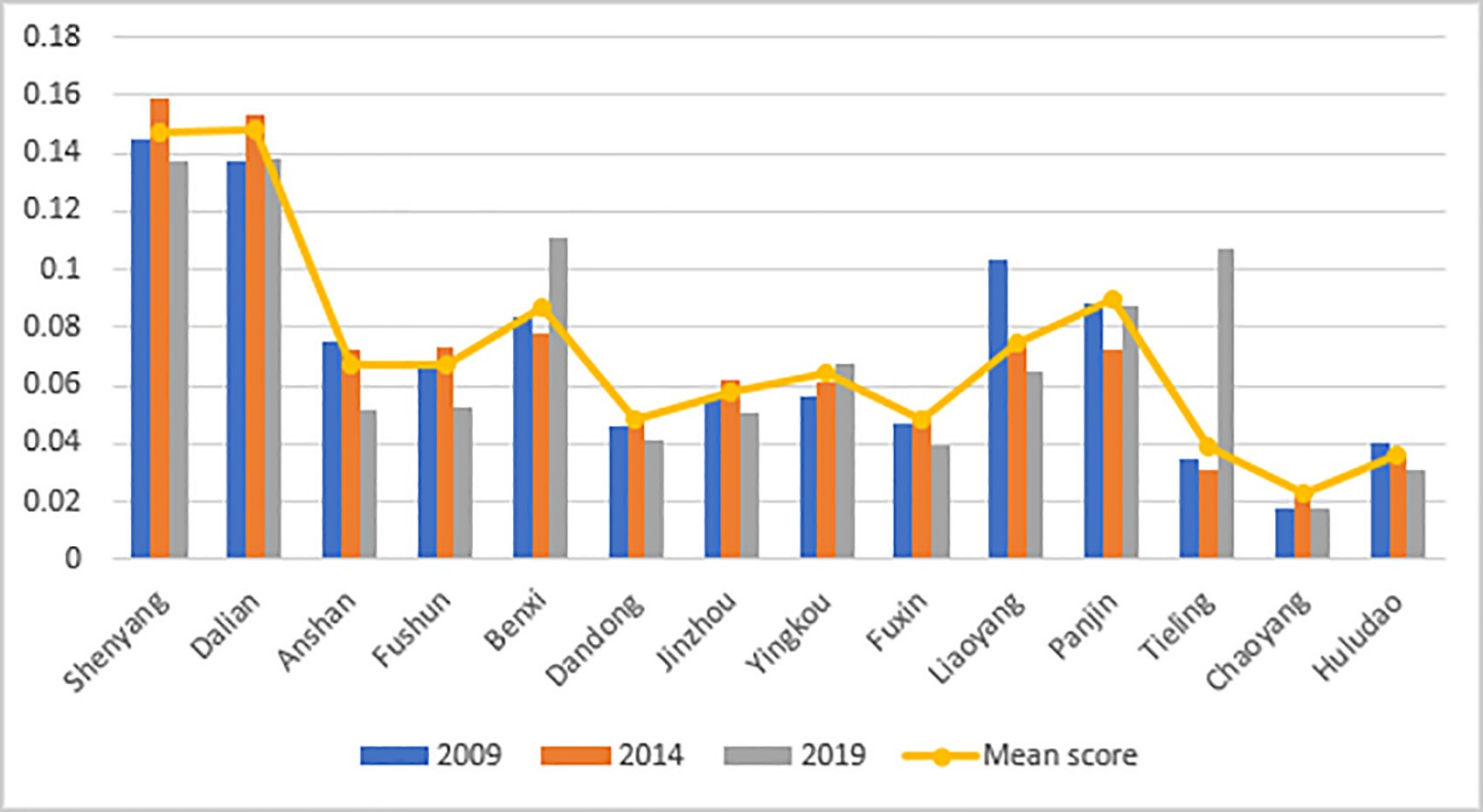
2009–2019 urban resilience score of Liaoning Province.

The urban resilience degrees of 14 cities in Liaoning Province from 2009 to 2019 were obtained through the comprehensive estimation and calculation of the data using the entropy method (Fig 2). Referring to the research of Zhang et al., [52] we divided the comprehensive urban resilience value (R) into three groups: high resilience, moderate resilience, and low resilience (Table 5).

**Table 5:** Types of urban resilience levels in Liaoning Province from 2009 to 2019.

Types of urban resilience levels	High resilience(R>0.12)	Moderate resilience(0.06≤R≤0.12)	Low resilience (R<0.06)
City	Shenyang, Dalian	Anshan, Fushun, Benxi, Yingkou, Liaoyang, Panjin	Dandong, Jinzhou, Fuxin, Tieling, Chaoyang, Huludao

Overall, the average urban resilience degree of Liaoning Province was 0.0714, which falls in the moderate group. The number of cities with high resilience accounted for 14.3% of the total number of cities, and the overall resilience degree was low. The regional spatial development was not balanced. During the study period, Dalian had the highest degree of urban resilience, with an average value of 0.1482; and Chaoyang had the lowest degree of urban resilience, with an average value of 0.0234. The gap in urban resilience showed a trend of “increasing first and decreasing later”, and the gap at the end was larger than that at the early stages, which indicated that a spatial “Matthew effect” was formed during the development of urban resilience in Liaoning Province in the early stage, but the “trickle-down effect” was more prominent in the later stage.

The characteristic of urban resilience in the cities of Liaoning Province may be described as “core-margin”. The core areas were Shenyang and Dalian, and the average urban resilience in these exceeded 0.12. These are areas with convenient external transportation, strong resource gathering ability, high linkage and coordination capabilities, and significant polarized positionality. However, due to the high degree of resilience, the overall variation was not obvious under the influence of the “ceiling effect”. The non-core areas included Anshan, Fushun, Benxi, Yingkou, Liaoyang, and Panjin, which all have a moderate degree of resilience. Most of them are resource-based cities and can resist certain risks. However, such cities still need to strengthen their own development to prevent resilience from falling back. The marginal cities are Dandong, Jinzhou, Fuxin, Tieling, Chaoyang, and Huludao, most of which are resource-exhausted cities, with inappropriate industrial structure, and in which the challenge of resource depletion and weak adjustment ability of the urban system have led to low degrees of urban resilience.

**Fig 3:**
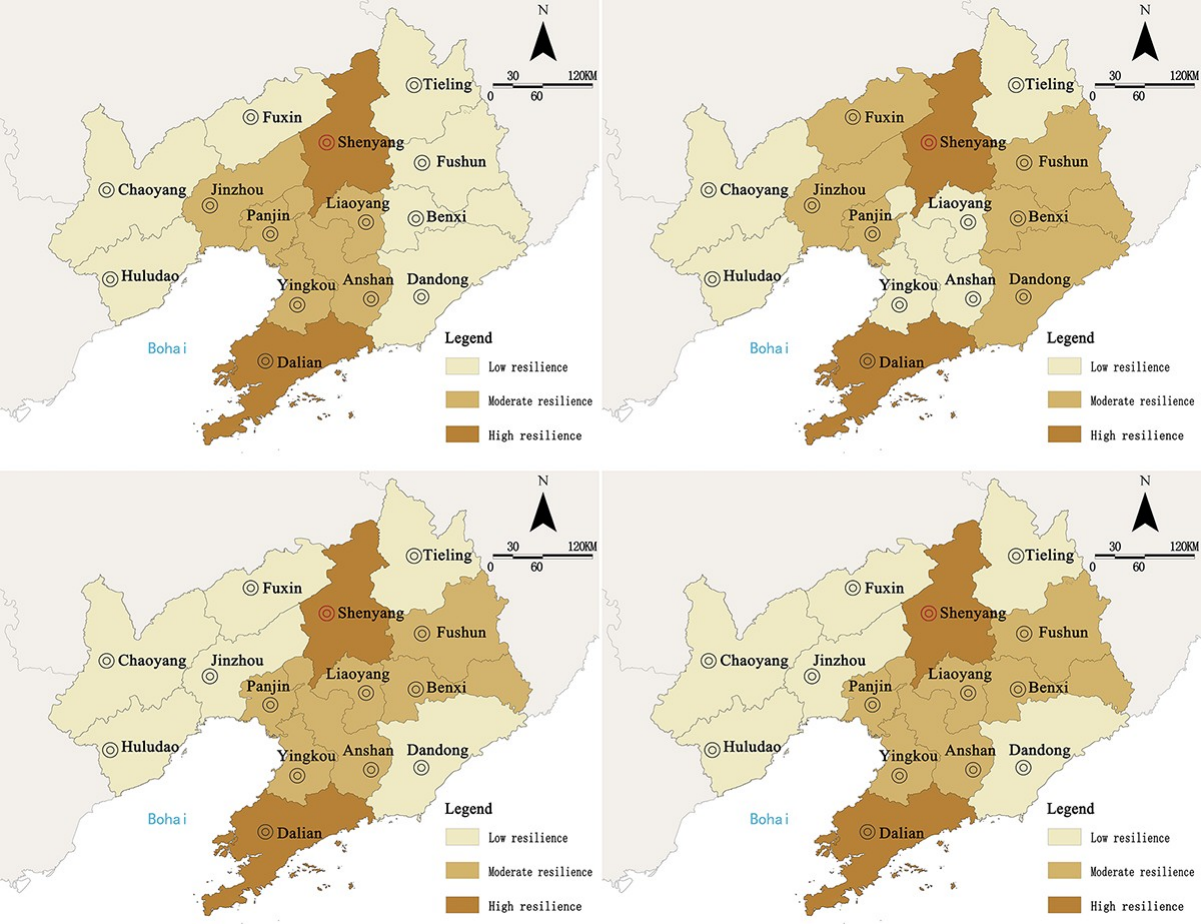
Spatial distribution of resilience of urban subsystems in Liaoning Province from 2009 to 2019. a. Economic resilience. b. Social resilience. c. Engineering resilience. d. Ecological resilience. (Source of base map: the open source map data service provided by the National Platform for Common GeoSpatial Information Services (https://www.tianditu.gov.cn/)).

Further analysis of the comprehensive score of subsystems showed that economic resilience and social resilience had the highest weightages as well as the greatest impact on urban resilience, which was followed by engineering resilience and ecological resilience. Among them, economic resilience and social resilience were in coordination with the overall variation trend in urban resilience, with obvious dual-core characteristics, which indicated that the degree of resilience was mostly affected by social development and industrial economy. From the perspective of space (Fig 3), Dalian and Shenyang were the areas with peak scores of subsystem resilience, and their degrees of economic development, social maturity, and infrastructure construction were higher than those of other cities. The spreading effect of outpost cities was significant, and an obvious correlation pattern with other cities was formed.

### 3.2 Differential characteristics of urbanization quality

**Fig 4:**
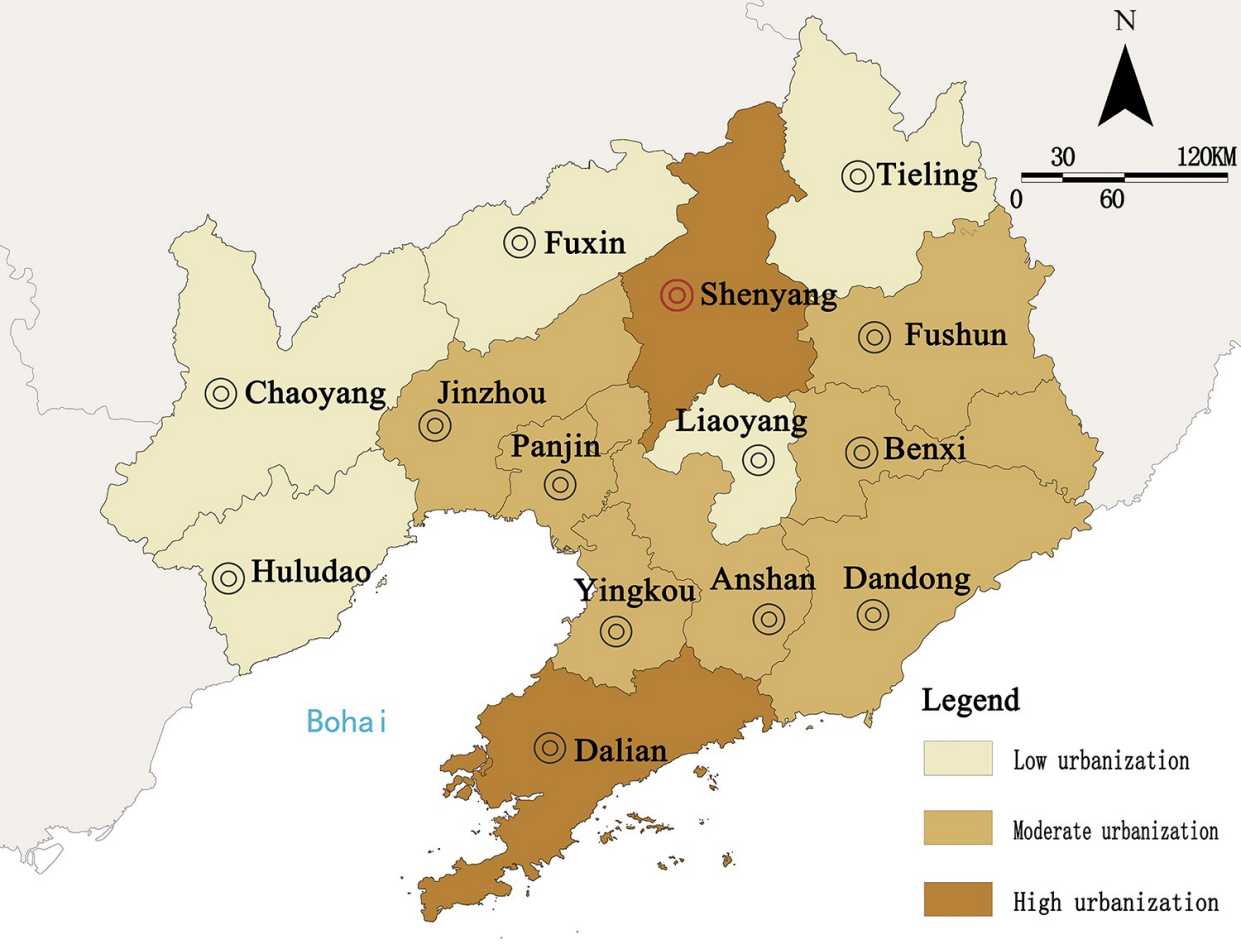
The spatial distribution of urbanization quality in Liaoning Province from 2009 to 2019 (source of base map: The open source map data service provided by the National Platform for Common GeoSpatial Information Services (https://www.tianditu.gov.cn/)).

The urbanization quality of 14 cities in Liaoning Province during the study period was evaluated using the entropy method. According to mathematical statistics and correlation, keeping the classification criteria range for each city as 0.5 times and 1.5 times of the average value of urbanization quality, the urbanization quality values were categorized into three levels: high urbanization, moderate urbanization, and low urbanization (Table 6), following which the spatial distribution was obtained (Fig 4).

**Table 6:** 2009–2019 Liaoning Province urbanization quality type.

Urbanization quality types	Highly urbanized (R>0.10)	Moderate urbanization (0.035≤R≤0.10)	Low urbanization (<0.035)
city	Shenyang, Dalian	Anshan, Fushun, Benxi, Jinzhou, Yingkou, Dandong, Panjin	Fuxin, Liaoyang, Tieling, Chaoyang, Huludao

Overall, the average score of urbanization quality in Liaoning Province increased from 0.0574 to 0.0966 and formed an upward fluctuating trend, and the quality of urbanization had improved. The reason was that in recent years, with the continued implementation of the Northeast Revitalization Strategy, Liaoning Province adjusted its industrial structure, strengthened its economic strength, and had its urbanization enter a stage of rapid development. However, problems such as the low utilization rate of land, wastage of resources, and outflow of skilled people have also affected the sustainable growth of urbanization.

From the perspective of spatial distribution, there were obvious “dual-core” characteristics of urbanization. With large population and industrial agglomeration capacity and high degree of urban development, Shenyang and Dalian formed the first echelon of urbanization. Anshan, Fushun, and other moderately urbanized cities accounted for the largest proportion. Relying on the “dual-core” trickle-down effect and resource advantages, these cities have made their way into the second echelon of urbanization development. Due to unreasonable urban industrial structure and resource depletion, cities such as Fuxin and Tieling had a lower degree of urbanization. Therefore, such cities should actively carry out industrial transformation, improve basic public service facilities, and promote the healthy development of cities and towns.

**Fig 5:**
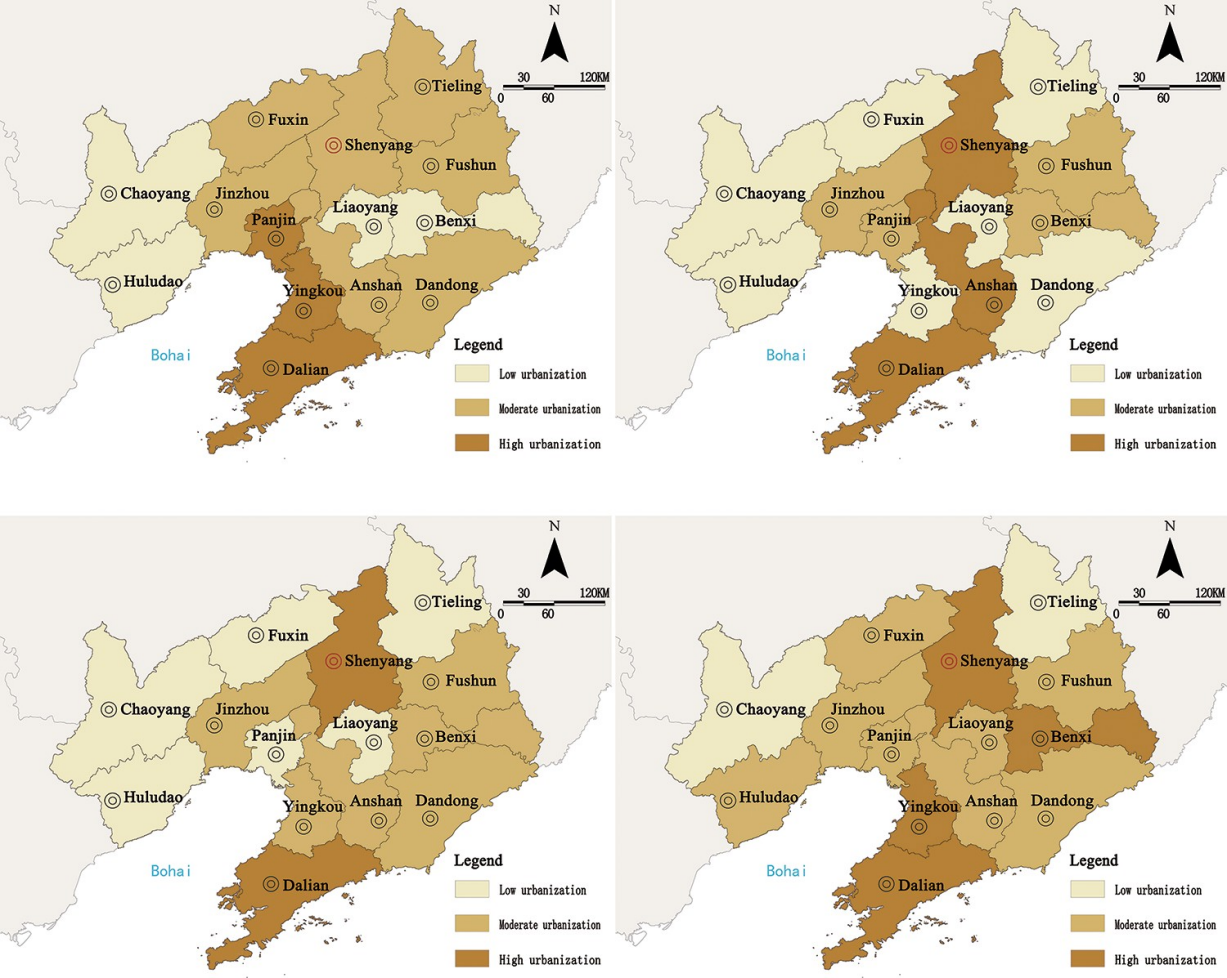
The spatial distribution of urban subsystem quality in Liaoning Province from 2009 to 2019. a. Population urbanization. b. Economic urbanization. c. Social urbanization. d. Land urbanization. (Source of base map: the open source map data service provided by the National Platform for Common GeoSpatial Information Services (https://www.tianditu.gov.cn/)).

In terms of each subsystem (Fig 5), there are obvious regional imbalance characteristics, and as the key power to drive the orderly development of urbanization, land urbanization and social urbanization accounted for the largest weightage. In spatial distribution, Shenyang and Dalian gained the highest scores and became the regional core growth pole with a significant “siphon” effect. The main reason is that the advantageous resources accumulated in relatively developed cities due to the profit-seeking nature of resources and the preferences in capital utilization. At the same time, the time-lag effect of industrial agglomeration also aggravated the differential level among regions.

### 3.3 Coupling analysis of urban resilience and urbanization quality

Based on the comprehensive scores of urban resilience and urbanization quality of 14 cities in Liaoning Province from 2009 to 2019, and by using the coupling coordination degree model, we calculated the coupling degree C and coordination degree D, and analyzed the characteristics of the coupling relationship.

#### 3.3.1 Spatial and temporal distribution characteristics

**Fig 6:**
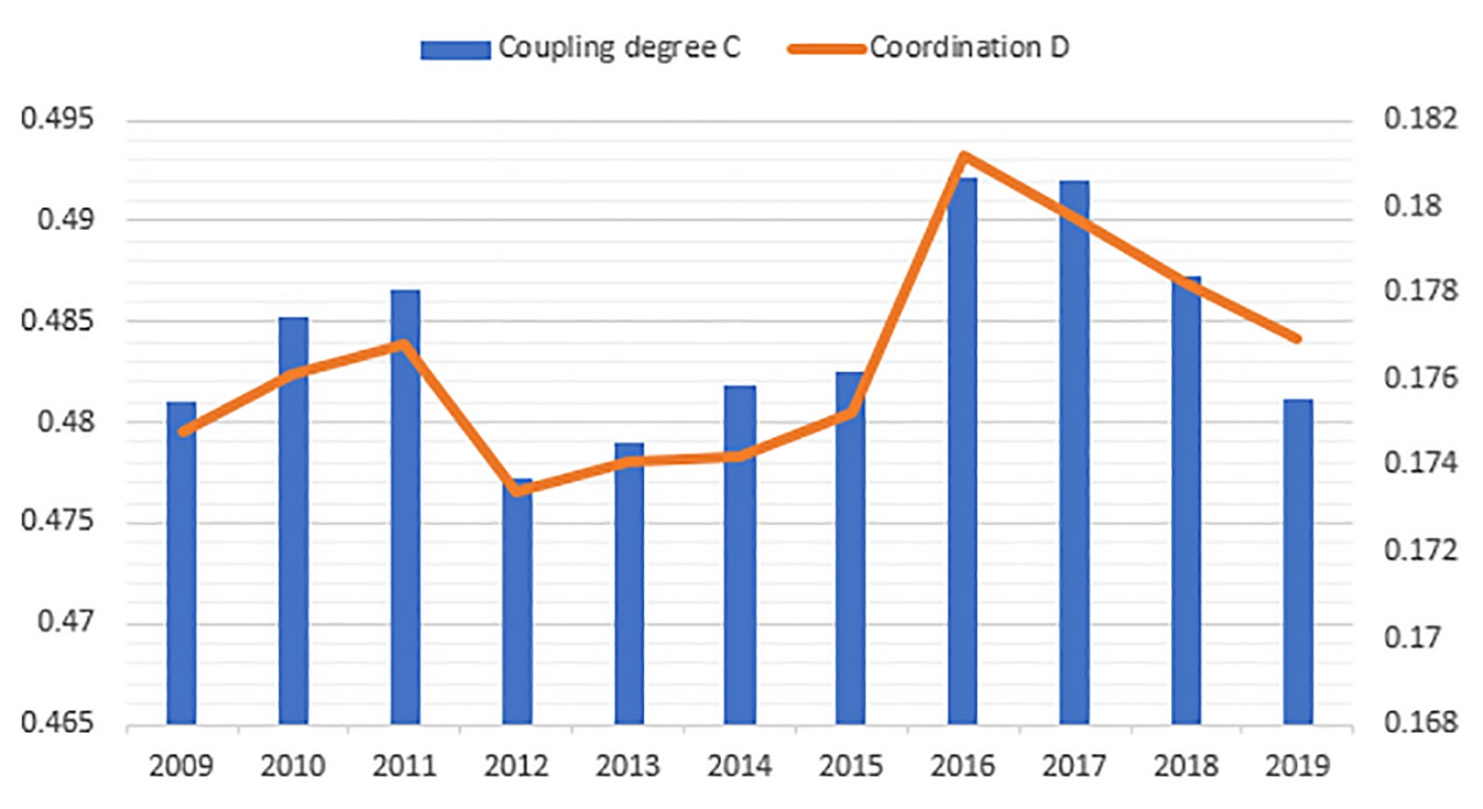
The evolution of urban coupling and coordination in Liaoning Province from 2009 to 2019.

From the variation trends in coupling degree and coordination degree of 14 cities in Liaoning Province (Fig 6), it can be seen that the coupling degree coefficient was higher than the coordination degree coefficient in the study period, and the two showed the same variation trend, which indicated that there was an interactive relationship between urban resilience and urbanization degree at this stage.

**Fig 7:**
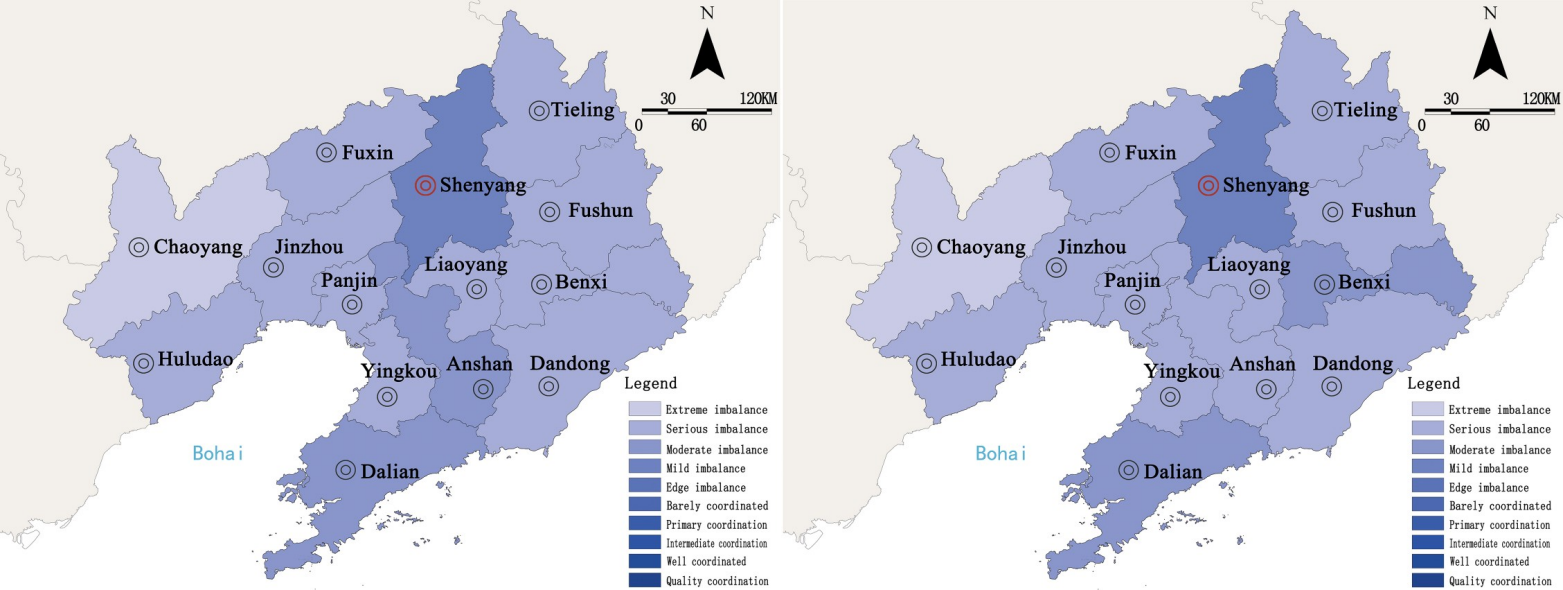
Spatial distribution of the degree of coupling and coordination of urban agglomerations in Liaoning Province in 2009 and 2019. a. 2009. b. 2019. (Source of base map: The open source map data service provided by the National Platform for Common GeoSpatial Information Services (https://www.tianditu.gov.cn/)).

This paper analyzed the degree of coupling coordination of cities in Liaoning Province between 2009 and 2019(Fig 7), and the results showed that the overall “urban resilience—urbanization quality” of Liaoning Province was in a maladjusted state, with unbalanced spatial distribution. In 2009, Shenyang had the highest coupling coordination degree, and was in a mild state of imbalance. Dalian and Anshan were in a moderate state of imbalance. The other 11 regions, such as Fushun and Benxi, were in a seriously unbalanced state, and Chaoyang City was in a state of extreme imbalance. There were several cities in the unbalanced state, and the overall coupling coordination degree was not high. The main reason was that the rapid development of urbanization in 2009 had put great pressure on the running of urban subsystems, and it was difficult to coordinate the degree of urban resilience and the development of urbanization.

By 2019, the overall coupling coordination degree of 14 cities in Liaoning Province had improved, with insignificant variation, although the 14 cities were still in a state of imbalance. Among them, Benxi improved from the state of serious imbalance to the state of moderate imbalance. The coordination degree of Anshan dropped from the state of moderate imbalance to the state of serious imbalance, while the imbalance state of Shenyang, Dalian, and the 12 other regions did not change. This might be due to the sluggish economic development, the depletion of urban resources, and the loss of population in Northeast China in recent years. The basic guarantee for urban resilience disappeared and the ability for self-regulation and recovery was weakened, therefore, it was difficult to improve urban resilience significantly.

#### 3.3.2 Temporal and spatial dynamic evolution of LISA

Using formula (8) and GeoDa1.12 spatial measurement software, we calculated the global Moran’s I for the coupling coordination degree of Liaoning Province’s urban agglomerations from 2009 to 2019 (Table 7).

**Table 7:** 2009–2019 Liaoning Province urban agglomeration coordination degree global Moran's I index.

	2009	2010	2011	2012	2013	2014	2015	2016	2017	2018	2019
Moran's	0.237	0.353	0.244	0.092	0.098	0.009	0.025	0.144	0.008	0.090	0.220
Z-Variance	1.908	2.953	1.971	0.555	0.892	0.178	2.030	0.438	0.187	0.832	1.733
p-value	0.023	0.001	0.021	0.297	0.186	0.403	0.024	0.231	0.446	0.214	0.007

The results showed that the Moran’s I of coupling coordination degree from 2009 to 2019 held a positive value in the range from 0.009 to 0.353. Among them, the values passed the 95% significance degree tests in 2009, 2010, 2011, 2015, and 2019, when the Moran’s I was 0.237, 0.353, 0.244, 0.025, and 0.220, respectively, which proved that the coupling coordination degree of urban resilience and urbanization quality were obviously positively correlated at this stage, and there was agglomeration effect in terms of space. However, in the years in which the significance degree test failed the coupling coordination degree tended to be randomly distributed with insignificant spatial correlation. The results showed that during the study period, the Moran’s I showed a downward trend in general, which indicated that the spatial agglomeration effect of coupling coordination degree between urban resilience and urbanization quality weakened.

**Fig 8:**
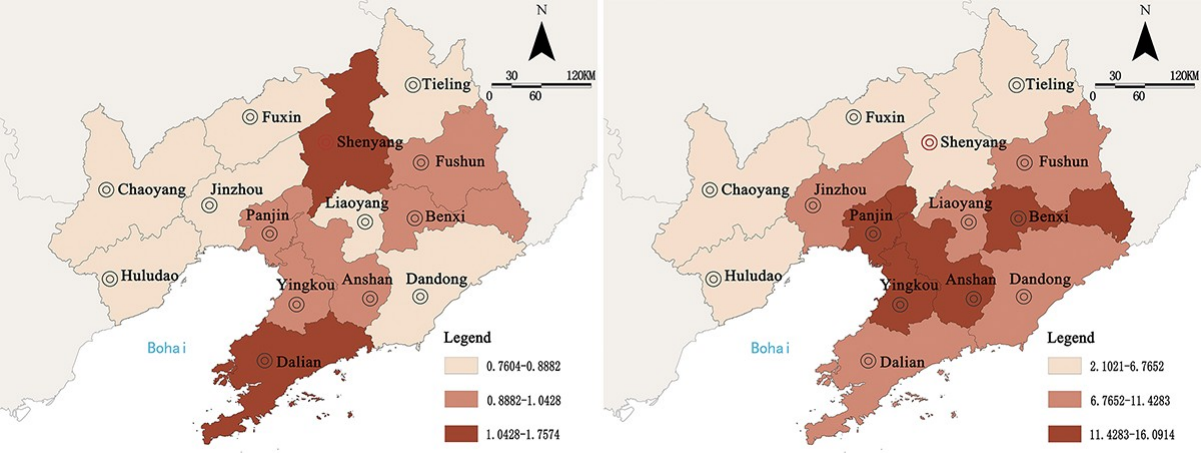
Spatial distribution diagram of geometric characteristics of LISA time path. a. Relative length. b. Curvature. (Source of base map: the open source map data service provided by the National Platform for Common GeoSpatial Information Services (https://www.tianditu.gov.cn/)).

In order to further understand the spatial-temporal linkage characteristics of the coupling relationship between urban resilience and urbanization quality in Liaoning Province, and by calculating the LISA time path, we obtained the relative lengths and curvature of the LISA time path movements of 14 cities in Liaoning Province from 2009 to 2019(Fig 8).

The relative lengths of LISA time path movement did not change much and presented a relatively dynamic local spatial structure. The spatial characteristics were prominent in the central region but were insignificant in the East and West sides of Liaoning Province, which indicated that Fuxin, Tieling, and other cities in the Eastern and Western sides developed at a slow speed due to backward infrastructure development and weak industrial foundation, which in turn lead to slow urbanization, insignificant changes in urban coupling coordination degree, and the formation of a stable local spatial structure; at the same time due to superior economic, industrial, and infrastructure development compared with those of surrounding cities, Shenyang, Dalian, and other cities in the central region experienced rapid development of urbanization with great variations in the coupling coordination degree, along with the local spatial structure showing dynamic characteristics.

The curvatures of LISA time path movements were all greater than 1, which indicated that the variation process of coupling coordination degree of 14 cities in Liaoning Province was fluctuating, exhibiting a distribution characteristic of “lower in the North and higher in the South” in general, wherein the space was clearly divided into two heterogeneous parts. This showed that urban resilience and urbanization quality development speed of Shenyang, Fuxin, and other northern cities had not changed significantly, that the coupling coordination degree was relatively stable, the variation of local spatial dependence direction was weak, and its spillover or siphon effect on surrounding cities was not obvious. The development speed of Anshan, Benxi, and other southern cities changed greatly, and in terms of spatial dependence the directions were toward adjacent areas; the spatial evolution process was fluctuating and dynamic, and was greatly affected by the spillover effect on adjacent cities.

## 4. Conclusion and discussion

### 4.1 Conclusion

Based on the panel data of Liaoning Province from 2009 to 2019, we studied the coupling relationship between urban resilience and urbanization quality for 14 cities in Liaoning Province using the coupling coordination degree model, and came to the following conclusions:

1. **Differential characteristics of urban resilience:** The number of highly resilient cities accounted for 14.3% of the total number of cities in Liaoning Province, and the degree of resilience was low in general; the spatial difference was significant, and showed a downward trend from the Shenyang and Dalian Economic Belt toward both sides, in which the average degree of resilience of Dalian and Shenyang was greater than 0.12 with significant “core-margin” characteristics. The evolutionary trend of subsystem development was: economic resilience > social resilience > engineering resilience > ecological resilience. As important outposts, Dalian and Shenyang presented prominent spreading effects, which can promote regional coordinated development.
2. **Urbanization quality:** From 2009 to 2019, the average score of urbanization quality in Liaoning Province increased from 0.0574 to 0.0966, and the urbanization quality showed a fluctuating upward trend; the spatial distribution varied among regions with prominent “dual-core” characteristics. The average urbanization quality score of Shenyang and Dalian was greater than 0.1, and these two cities were highly urbanized, had strong economic strength and concentration of resources, and had become the regional core growth pole with a significant “siphon” effect. Having been given bigger weightage, land urbanization and social urbanization were the key driving forces to improve the quality of urbanization.
3. **Coupling coordination analysis:** From the perspective of coupling coordination, quantitative analysis and spatial analysis of urban resilience and urbanization quality. According to the analysis of quantitative indicators, from 2009 to 2019, 14 cities in Liaoning Province were in a state of imbalance, and there was a positive correlation between the spatial coupling degree and the coordination degree, however, the Moran's I decreased from 0.237 to 0.220, there is a downward trend, indicating that the spatial agglomeration feature has weakened. There were significant differences in spatial distribution, forming a pattern with Shenyang and Dalian as the core, and areas with low coupling degree being distributed in the shape of a circle. Through further analysis of the spatial distribution characteristics, it is concluded that the relative length of the LISA time path exhibits the spatial characteristics of convex in the middle of Liaoning Province and contraction on the east and west sides. The curvature of the LISA time path movement is greater than 1, and shows a characteristic distribution, that is, lower in the north and higher in the south.

### 4.2 Discussion

#### 4.2.1 Innovation potential

As the core subject of sustainable science, urban resilience is also a frontier subject in the study of new-type urbanization and has received extensive attention from many subjects. As an organic whole, the construction of urban adaptability is closely related to the quality of urbanization. Here have been several studies on the comprehensive evaluation of urban resilience [53], or urbanization and development of urban subsystems, but fewer studies on the coupling relationship between urban resilience and urbanization. Compared with the research on urban disaster prevention capability from a single perspective such as infrastructure [54], landscape pattern [21] and urban ecology [55], grasping and analyzing the interaction logic of the interaction between the two from a coupling perspective can explain the comprehensiveness and complexity of urban development more scientifically. It provides a theoretical basis for coordinating the relationship between urban population and land.

Furthermore, existing research data are limited to social statistical data [56, 57]. However, the research in this paper was based on both physical geography data and social statistics data, and studied the coupling relationship between resilience degree and urbanization quality of 14 cities in Liaoning Province, which makes it forward-looking to some degree.

The spatial distribution of urban resilience and urbanization quality differs. According to existing studies, with the increase in coupling coordination degree between urban resilience and urbanization quality of urban agglomerations, the differences between them are gradually narrowed, and the spatial agglomeration is gradually strengthened [52]. The degree of coupling coordination of 14 cities in Liaoning Province were analyzed in this paper, and the degree of overall coordination between urban resilience and urbanization quality increased from 2009 to 2019, showing a positive correlation, however, the spatial agglomeration effect of urban agglomeration weakened, which is a different outcome from the research results of other scholars.

During the study period, the spatial agglomeration of 14 cities in Liaoning Province has declined, and the regional coordination between cities is poor. In the future development of Liaoning Province, the concept of resilience should be incorporated into the development plan, and the regional development speed should be coordinated to form a mutually beneficial and complementary development pattern. So as to efficiently promote the coordinated development of cities in Liaoning Province. For fast-developing cities such as Shenyang, it is necessary to actively play a core and leading role, radiate small cities from big cities, promote the free flow of regional production factors, and effectively balance resource differences between cities; for small and medium-sized cities such as Chaoyang, it is urgently needed Accelerate transformation and development, improve urban safety measures, improve risk management, and enhance regional risk prevention capabilities.

#### 4.2.2 Limitations

Through the coupling analysis of the urban resilience and urbanization quality of 14 cities in Liaoning Province from 2009 to 2019, this paper provides a reference data framework for improving the quality of urbanization and promoting regional coordinated development. However, this paper still has the following limitations: 1. The statistical data obtained in this paper may be slightly different from the real situation due to the differences in statistical quality of the sources; 2. The ground resolution of satellite remote sensing images is not completely unified, and the statistical information of land boundaries may have slight deviations from the real situation; 3. Considering the availability of data, the difference between the selected indicator data and the grading threshold value for evaluation is not completely identical.
